# Long-term outcomes of platelet-rich fibrin and blood clot scaffolds in regenerative endodontic treatment of traumatized immature maxillary incisors: An 80-month case report

**DOI:** 10.3389/fdmed.2026.1894791

**Published:** 2026-08-03

**Authors:** Sevgi Arabulan, Özant Önçağ, Nesrin Dündar, Nazan Ersin, Cemal Eronat

**Affiliations:** 1 Department of Conservative Dentistry and Periodontology, University Hospital Regensburg, Regensburg, Germany; 2 Department of Pediatric Dentistry, Faculty of Dentistry, Ege University, Izmir, Türkiye; 3 Independent Consultant in Oral and Maxillofacial Radiology, Boston, MA, United States

**Keywords:** dental pulp necrosis, disinfection, platelet-rich fibrin, regenerative endodontics, reinfection, revitalization, tissue engineering

## Abstract

Regenerative endodontic procedures offer a biological treatment for immature permanent teeth after pulp necrosis, enabling root maturation and return of sensitivity. Platelet-rich fibrin (PRF) has been suggested as a biologically advantageous scaffold, although evidence comparing it with the traditional blood clot (BC) remains limited. The BC-treated tooth developed reinfection and required retreatment at 3 months, limiting direct comparison between the two scaffolds. Early outcomes favored PRF, with greater root maturation and apical closure within the first 12–28 months, while the BC-treated tooth showed partial closure and canal obliteration. However, the long-term outcomes differed markedly at the 80-month follow-up: PRF-treated tooth lost sensibility and developed an apical radiolucency, but the BC-treated tooth showed no apical pathology, and maintained an early positive sensibility response. Although direct conclusions cannot be drawn from this single case, the findings suggest that early radiographic signs of maturation may not necessarily predict long-term stability and underscore the importance of secondary infection and extended follow-up in regenerative endodontic treatment.

## Introduction

Immature permanent teeth with necrotic pulps pose a significant endodontic challenge due to their immature roots with open apices and thin root canal walls. Traditionally, management has relied on apexification with long-term calcium hydroxide or an MTA apical plug, which creates an apical barrier but does not promote further root development, leaving teeth susceptible to fracture (1, 2). In contrast, revitalization aims to re-establish vitality and continue root maturation by inducing periapical bleeding to form an intracanal blood clot rich in stem cells and growth factors (3, 4).

Platelet-rich fibrin (PRF), an autologous platelet concentrate, have been proposed as scaffold provides a stable fibrin matrix with sustained release of bioactive molecules, supporting angiogenesis, neurogenesis, and odontoblastic differentiation (5, 6).

Although some studies suggest that PRF may improve root development and reduce the risk of crown discoloration (7, 8), the clinical evidence is inconclusive, with comparable success for PRF and BC in terms of periapical healing, apical closure and survival of the treated tooth (9, 10).

However, regenerative endodontic procedures remain associated with several limitations, including variable radiographic outcomes and uncertainty regarding long-term stability (11). Evidence beyond five years is still limited, although late complications such as recurrent infection, inflammatory root resorption, coronal microleakage, pulp canal obliteration, or loss of sensibility response may emerge only after prolonged follow-up (12, 13). Therefore, long-term studies, as well as case reports are valuable for clarifying the potential limitations of regenerative endodontic treatment.

This case report describes regenerative endodontic treatment of two traumatized immature maxillary incisors in a 6-year-old child, using two different scaffold approaches, blood cloth and PRF, over an 80 months follow-up period. By documenting long-term outcomes in the same patient, this report aims to contribute to the limited evidence on the long-term stability of regenerative endodontic treatment.

## Case description

This case report follows Case Report (CARE) guidelines (14), and written informed consent for diagnostic, treatment, and publication purposes was obtained from the patient's guardians. Institutional policy does not require ethics approval for single case reports.

**Figure 1:**
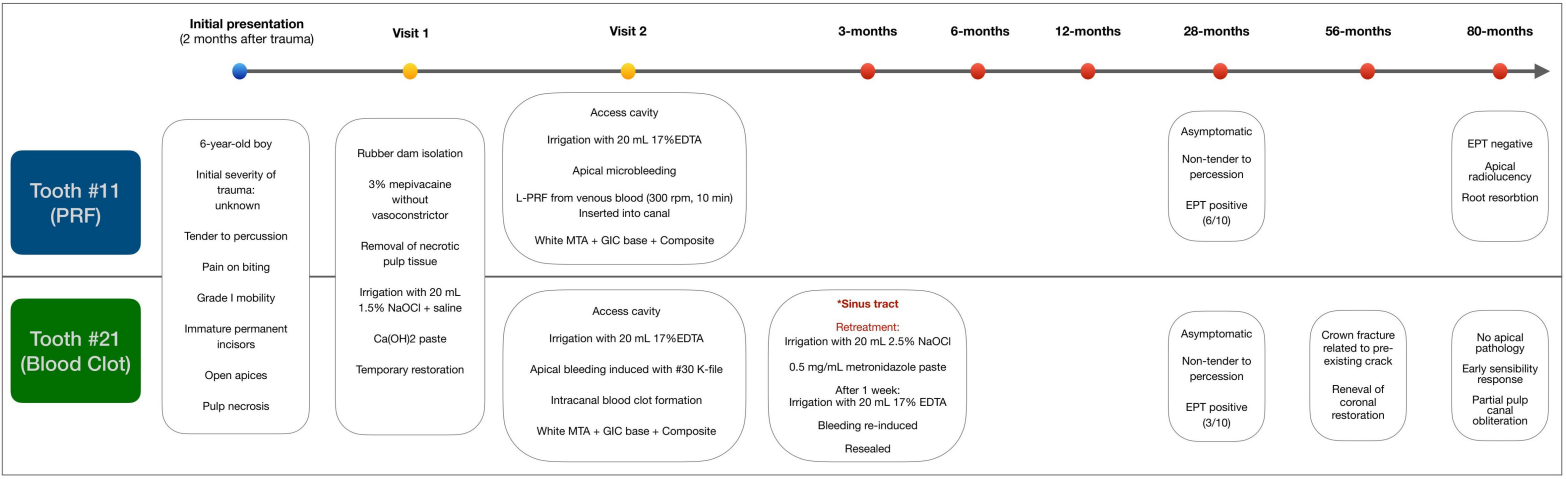
Timeline of the diagnostic findings, regenerative endodontic procedures, retreatment, and follow-up outcomes of teeth #11 and #21. Tooth #11 was treated using platelet-rich fibrin (PRF), whereas tooth #21 was treated using a blood clot (BC) scaffold.

A 6-year-old boy presented two months after trauma to the maxillary central incisors. Since the patient did not seek acute care following the trauma, the type and extent of the injury remain unknown. This should be considered a descriptive within-patient case observation rather than a controlled comparison of scaffold materials, because two teeth subsequently followed different clinical courses, including retreatment of tooth #21 after early signs of infection with different medicament. The overall treatment sequence and long-term follow-up outcomes are summarized chronologically in Figure 1.

**Figure 2:**
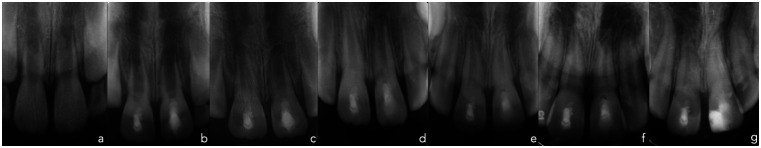
Serial periapical radiographs showing the radiographic follow-up of the immature maxillary central incisors after regenerative endodontic treatment. **(a)** Pre-operative, **(b)** 1 month, **(c)** 3 months, **(d)** 6 months, **(e)** 12 months, **(f)** 28 months, and **(g)** 80 months.

Clinical examination revealed tenderness to percussion, a complaint of pain when biting and Grade I mobility on tooth #11 and #21 (Figure 2a). Due to pulp necrosis, regenerative endodontic procedures were planned: Tooth #21 was assigned to revitalization with a blood clot (BC), and tooth #11 to a PRF scaffold placed after induction of apical microbleeding.

All procedures were performed under rubber-dam isolation and 3% mepivacaine without vasoconstrictor. Access cavities were prepared, necrotic pulp removed, and canals irrigated with 20 mL 1.5% sodium hypochloride (NaOCl) followed by same amount of saline, then dried. Calcium hydroxide was placed in the coronal third and cavities temporarily sealed. After confirming the absence of symptoms, at the second visit, canals were re-accessed, and irrigated with 17% EDTA. In tooth #21, apical bleeding was induced by extending the #30 K-file 2 mm beyond the apex to form a blood-clot scaffold.

In #11, venous blood was collected and processed according to the L-PRF protocol (3 000 rpm, 10 min) (15), the fibrin clot gently compressed (16), sectioned and inserted into the canal after microbleeding. Both teeth were then sealed with white MTA and restored with glass ionomer base and composite (Figure 2b). At three months, #21 developed pain on percussion and a sinus tract; MTA and restoration were removed, the canal disinfected with 20 mL 2.5% NaOCl, dressed with 0.5 mg/mL metronidazole paste, after one week irrigated with 20 mL of 17% EDTA, followed by bleeding induction and MTA/composite sealing.

**Figure 3:**
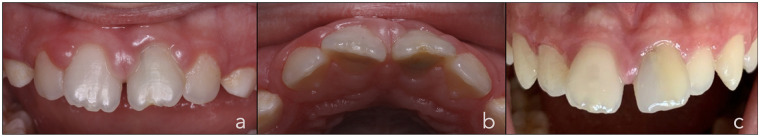
Intraoral view. **(a)** Frontal view of tooth 11 and 21 at 28th month. **(b)** Occlusal view at 28th month. c) Frontal view at 80th month.

During follow-up, tooth #21 (BC-treated) showed radiographic changes consistent with root maturation (thickening of the canal walls, continued root development). Periapical radiographs showed early root dentinal wall thickening at 3 months (Figure 2c), more advanced maturation at 6 months (Figure 2d), and superior apical closure at 12 months (Figure 2e), whereas tooth #11 (PRF-treated) displayed partial closure and canal narrowing. However, these findings were assessed qualitatively, as precise standardization of all periapical radiographs using a parallel technique was not feasible because of limited patient cooperation. At the 28-month review (Figures 2f, 3a,b), both incisors were asymptomatic, non-tender, and responded to electric pulp testing, with a lower threshold in the BC tooth (3/10) than in the PRF tooth (6/10). At the 56-month, patient a crown fracture related to a pre-existing crack was observed in tooth #21 and tooth was restored with composite resin in another institution.

At the 80-month follow-up, both teeth were tender to percussion, and tooth #21 exhibited crown discoloration (Figures 2g, 3c). Electric pulp testing indicated that tooth #11 has no response to sensibility test, whereas tooth #21 produced an early sensibility response (19/64).

**Figure 4:**
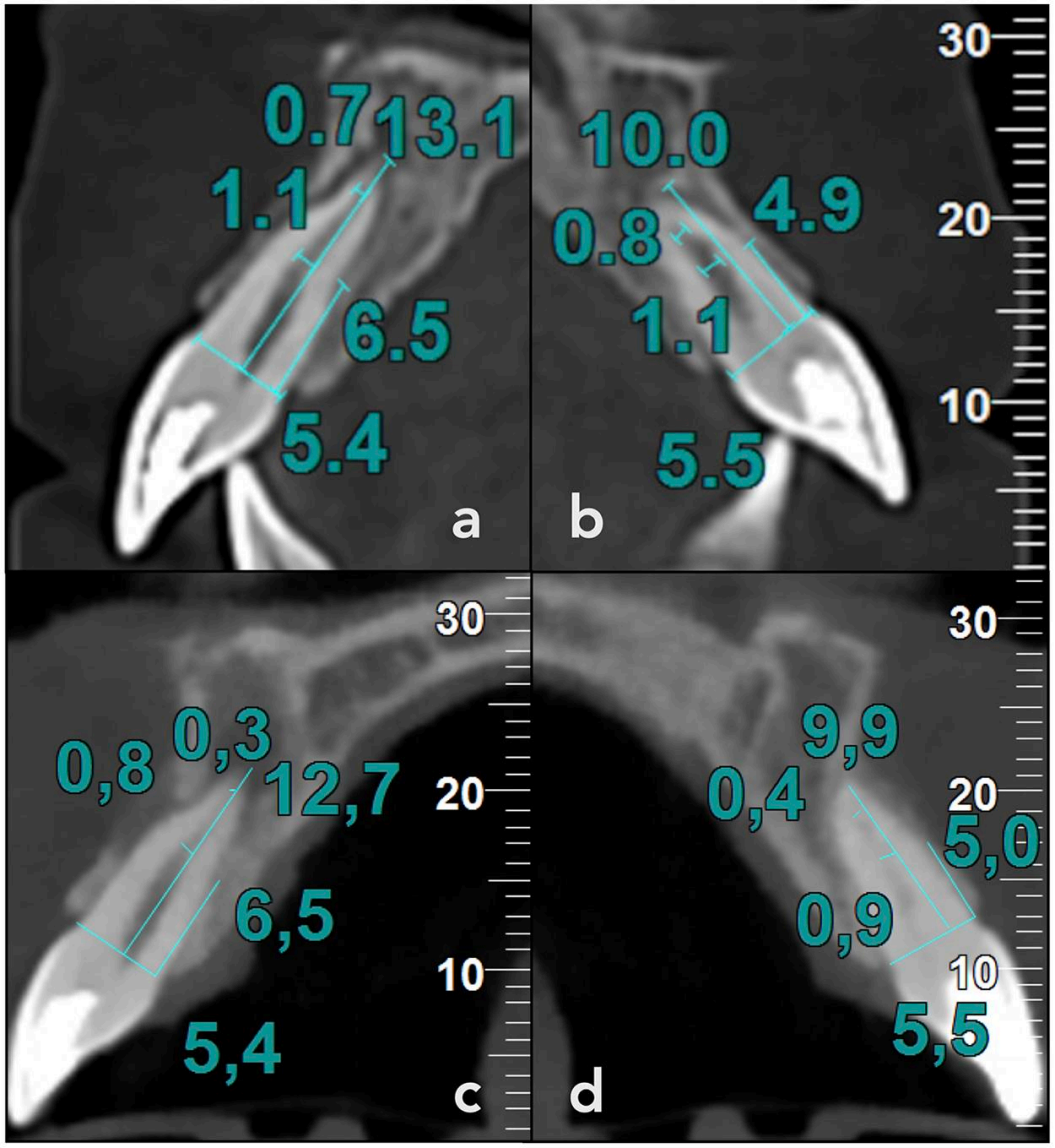
Comparison of CBCT images of PRF and BC-treated teeth. **(a)** Tooth #11 at 28-month (PRF). **(b)** Tooth #21 at 28-month (BC). **(c)** Tooth #11 at 80-month (PRF). **(d)** Tooth #21 at 80-month (BC).

Linear measurements of root morphology and pulp space dimensions were obtained from cone.beam computed tomography (CBCT) images at predefined root levels at the 28- and 80-month follow-ups (Figure 4).

CBCT images were acquired using a NewTom VGi evo CBCT unit (Cefla Medical North America) with a 5 × 5 cm field of view (FOV) to evaluate the maxillary anterior region. Images were obtained with the manufacturer's automatic exposure control (SafeBeam™), which automatically adjusts tube voltage, tube current, and exposure according to the selected acquisition protocol with the 0,2 mm voxel size and patient characteristics. Images were exported in DICOM folder for subsequent analysis. Image processing and analysis were performed using NNT Viewer Software (NNT Medical Suits, EU Regulation 2016/679). In the coronal plane, with a slice thickness of 1 mm, the approximate dimensions of the periapical lesion were measured as 3.3 × 2.1 × 3.2 mm.

Teeth number 11 and 22 were measured from the dentinoenamel junction to the apical end. Then, the pulpal canal width was measured in the area corresponding to half of this length and 2 mm above the apical opening (Figures 4a,b). 52 months later, these measurements were repeated on both teeth using the same points as a guide (Figures 4c,d). All measurements were conducted by an oral and maxillofacial radiologist more than 30 years of clinical experience. All measurements were performed on the same 15-inch Lenovo PC computer (Lenovo United States Incorporated 1,009 Think Place - Building One Morrisville, NC 27560 U.S.A.) with a resolution of 1,920 × 1,080 pixels and 10-bit colour support. To assess intraobserver agreement, all measurements were repeated 2 weeks after the initial evaluation was completed.

At the 80-month follow-up, partial pulp obliteration in both teeth, more prominently on tooth #21. In tooth #11, root resorption was observed with a wide periapical lesion, while the contralateral tooth was stable.

## Discussion

This case presents two treatment modalities for the revitalization of immature permanent teeth. After revitalization, the tissue formed typically represents functional repair rather than true pulp regeneration (17). To overcome the limitations of reparative tissue formation, biomimetic scaffolds such as PRF have been intoduced. PRF provides sustained release of growth factors that support stem-cell differentiation (5). Findings from the broader endodontic literature on autologous platelet concentrates may provide potential benefits for postoperative discomfort and radiographic healing, although the authors emphasized that heterogeneity and risk of bias prevent definitive conclusions regarding clinical effectiveness (18). Consistently, the evidence comparing PRF with other scaffolds in regenerative endodontic treatment remains limited and partly conflicting. While Ulusoy et al. reported superior root maturation and fewer complications with PRF compared with BC and PRP (7), Lv et al. found no significant differences between PRF and BC, noting that their protocol did not include deliberate apical microbleeding prior to PRF placement (19).

This case report describes a clinical scenario involving two traumatized immature maxillary incisors in the same pediatric patient, treated with different scaffold approaches and followed for 80 months. Although the case offers an opportunity to observe divergent long-term outcomes within the same patient, differences in baseline conditions and treatment course preclude a direct comparative interpretation. An important limitation of this case is that the original traumatic injury was not diagnosed at the acute stage. Thus, potential differences in the initial severity of trauma may have influenced the different long-term outcomes of the two teeth. Moreover, their treatment courses were not identical, since the BC-treated tooth required retreatment three months later due to infection, involving a modified disinfection protocol with a higher NaOCl concentration and the use of an intracanal antibiotic paste. Since disinfection is a critical determinant of regenerative endodontic treatment outcomes, these protocol differences represent major confounding factors and prevent the observed outcomes from being attributed solely to the scaffold material.

During the first 28 months, the PRF-treated tooth demonstrated more pronounced root development, while the BC-treated tooth showed partial apical closure and canal narrowing consistent with mineralized tissue formation. Although these findings are consistent with the reported biological properties of PRF, including the gradual release of bioactive molecules involved in cellular differentiation and mineralization (20), the observed differences cannot be attributed solely to the scaffold material because of the differing clinical courses and treatment protocols. While platelet-concentrates are considered biologically promising (21, 22), the overall quality of evidence remains moderate to low due to methodological differences across studies (23), emphasizing the need for more rigorous comparative research. However, the extended follow-up over 6 years revealed a contrasting long-term pattern. The PRF-treated tooth developed an apical radiolucency and loss of sensibility, while the BC-treated tooth showed no apical radiolucency and remained responsive to testing despite intracanal obliteration and a cervical restoration defect. Given the tooth-specific differences in clinical course and retreatment history, these outcomes cannot be interpreted as a direct comparison of scaffold performance. Nevertheless, the divergent long-term findings highlight that early radiographic signs of root maturation, even when clinically encouraging, may not necessarily correspond to long-term stability. Similarly, sensibility test responses after regenerative endodontic procedures should be interpreted with caution. A positive response to electric pulp testing does not necessarily indicate true pulp regeneration, as neural elements may develop independently from complete pulp tissue regeneration and false-positive responses remain possible. Conversely, loss of sensibility does not automatically imply complete loss of vitality. Therefore, in the present case, sensibility findings were considered supportive clinical observations rather than direct indicators of the histological status of the pulp-like tissue.

Pulp canal obliteration represents an important but controversial outcome after regenerative endodontic procedures (24, 25). While it may be associated with continued hard tissue deposition, it does not necessarily indicate true pulp regeneration and may complicate future retreatment if needed. In the present case, these findings further support the need for long-term follow-up after regenerative endodontic treatment.

Secondary infection is a major threat to the long-term success of regenerative procedures (26). As an early complication, the BC-treated tooth developed clinical signs of infection three months post-operatively. To improve bacterial control (27), NaOCl concentration was increased from 1.5% to 2.5% at the retreatment visit (3, 4). Even with stronger irrigation, residual microbes may persist, triggering chronic inflammation and uncontrolled intracanal mineralisation (12). Consistenly with a study suggesting scaffold choice can influence hard-tissue deposition (28), intracanal obliteration was markedly greater in the BC-treated tooth in long term. In contrast, despite the absence of visible restoration defects in the PRF-treated tooth, a large apical radiolucency was observed at long-term follow-up, suggesting that secondary infection, potentially facilitated by trauma-induced microcracks or late coronal microleakage, may have been more critical for prognosis than scaffold choice (29).

Another important consideration in pediatric dentistry is patient cooperation, particularly during multi-visit or invasive procedures. Although PRF may offer early biological advantages, long-term data remain limited, and its use requires venous blood collection, which can distress young children, reduce compliance and also influence parental acceptance of procedure. In contrast, a naturally formed blood clot eliminates this step, simplifying the procedure. Therefore, scaffold selection should take into account the child's behavior, parental consent and preferences, anxiety level, and overall treatment management.

## Conclusion

In this long-term case, both scaffolds initially supported periapical healing and root maturation, but their outcomes diverged over 6 years. The PRF-treated tooth eventually developed apical radiolucency and loss of vitality, whereas the BC-treated tooth remained without apical pathology despite marked intracanal obliteration. Rather than supporting the superiority of one scaffold over another, this case highlights the potential importance of secondary infection, possibly related to trauma-induced microcracks, in long-term prognosis. However this observation should be interpreted with caution. Additionally, scaffold selection should be individualized in pediatric patients, balancing biological potential and patient cooperation, always accompanied by long-term follow-up.

## Data Availability

The original contributions presented in this case report are included in the article. Further inquiries can be directed to the corresponding author.
